# Risk factors for human cutaneous anthrax outbreaks in the hotspot districts of Northern Tanzania: an unmatched case–control study

**DOI:** 10.1098/rsos.180479

**Published:** 2018-09-05

**Authors:** Elibariki R. Mwakapeje, Sol Høgset, Adis Softic, Janneth Mghamba, Hezron E. Nonga, Robinson H. Mdegela, Eystein Skjerve

**Affiliations:** 1 Epidemiology and Diseases Control Section, Ministry of Health, Community Development, Gender, Elderly and Children, PO Box 9083, Dar es Salaam, Tanzania; 2 Department of Veterinary Medicine and Public Health, Sokoine University of Agriculture, PO Box 3021, Chuo Kikuu Morogoro, Tanzania; 3 Faculty of Veterinary Medicine, Norwegian University of Life Sciences, PO Box 8146 Dep., 0033 Oslo, Norway

**Keywords:** cutaneous anthrax, cultural practices, animal and human interactions, Tanzania

## Abstract

*Bacillus anthracis* is an aerobic, Gram-positive and spore-forming bacterium, which causes anthrax in herbivores. Humans get infected after coming into contact with infected animals' products. An unmatched case–control study was conducted to identify the importance of demographic, biological and/or behavioural factors associated with human cutaneous anthrax outbreaks in the hotspot areas of Northern Tanzania. A semi-structured questionnaire was administered to both cases and controls. The age range of participants was 1–80 years with a median age of 32 years. In the younger group (1–20 years), the odds of being infected were 25 times higher in the exposed group compared to the unexposed group (OR= 25, 95% CI = 1.5–410). By contrast, the odds of exposure in the old group (≥20 years) were three times lower in the exposed group compared to the unexposed group (OR = 3.2, 95% CI = 1.28–8.00). Demographic characteristics, sleeping on animal's skins, contacting with infected carcasses through skinning and butchering, and not having formal education were linked to exposure for anthrax infection. Hence, a One Health approach is inevitable for the prevention and control of anthrax outbreaks in the hotspot areas of Northern Tanzania.

## 1. Introduction

*Bacillus anthracis* is an aerobic, gram-positive and spore-forming bacterium belonging to the family Bacillaceae [1]. The release of this bacterium (the causative agent for anthrax) from a dead infected host into the environment induces spore formation [2], enhancing the agent's ability to survive in the soil for a long time [3]. Despite being well controlled in developed countries, anthrax continues to have a devastating global effect on the poor and marginalized populations that depend on small-scale livestock farming in rural areas [4]. Anthrax is continuously ranked as a significant poverty-related neglected zoonotic disease, defined by the World Health Organization as a disease that ‘perpetuate poverty by affecting not only people's health but also their livelihoods’ [4,5]. Flooding, drought and biological vectors (birds, insects or scavengers) or areas of temporary stagnant water may exacerbate anthrax outbreaks [6]. The release of *B. anthracis* from an infected host into an aerobic environment with insufficient nutrients to sustain bacterial replication induces sporulation [7]. The *B. anthracis* spores are resistant to extreme conditions such as pH [8], heat, cold, desiccation and chemical agents, and may, in specific environments, survive up to 200 years [9]. Owing to the extended persistence of *B. anthracis* spores in the environment, regular epidemics may occur after a long time, such as a recent outbreak in Sweden after 27 years [10].

Anthrax affects all mammals, but wild and domesticated herbivorous dominate the numbers, as they are often infected through ingestion or inhalation of spores while grazing [11]. The susceptibility to infection differs depending on the host species [12], with cattle and sheep being the most vulnerable species followed by goats, dogs and horses [13]. Humans are considered to have a moderate susceptibility, while pigs and carnivores are more resistant [11]. Upon ingestion, spores enter macrophages of a susceptible host and are transported to lymph nodes where they germinate into vegetative form [14] and migrate into the bloodstream and release toxins which cause systemic effects [11].

Humans typically get infected with *B. anthracis* through oral, cutaneous and respiratory routes [15], and the infection could occur during direct contact when butchering, eating raw or undercooked meat, or handling products from infected animals [16]. Cutaneous anthrax is the most frequently diagnosed form of the disease in humans and occurs within 2–6 days after direct contact with anthrax spores [17]. It presents as a papular to a vesicular ulcer which forms a depressed black eschar which is accompanied by oedema [18].

The first anthrax outbreak in Tanzania was documented among the wildlife species in the national parks during 1962–1998, causing the death of 1200 impalas, and posed a great risk to humans and susceptible livestock [19]. Later on, sporadic human cases have been reported in different parts of the country. In 1985, a total of 239 human anthrax cases were reported in the Rukwa valley in southwest of Tanzania [20], and in 1988, a total of 11 human cases of cutaneous anthrax were admitted and treated at Mvumi Hospital in the Dodoma region of central Tanzania after patients came into contact with the infected animal carcasses [21].

In 1985, hundreds of different species of wildlife carcasses were laboratory-confirmed to have died from anthrax in the Selous game reserve [22], and in 1988, a big anthrax outbreak in wildlife was reported in the Tarangire national park in which 142 impalas, three zebras, four wildebeests and one giraffe were counted dead [23]. Since then, different species of wildlife and livestock and humans have frequently been affected by *B. anthracis*, with varying disease patterns between years in terms of the size of outbreaks and species affected [24].

Anthrax is a notifiable zoonotic disease in Tanzania, and it is a disease of public and animal health importance [16]. Despite the seriousness of anthrax outbreaks in animals, there is a poor surveillance system in the animal sector leading to under-reporting of reportable diseases [25], including anthrax.

Moreover, episodes of anthrax outbreaks are increasingly becoming a threat to humans, livestock and wildlife in Northern Tanzania, specifically in the Arusha and Kilimanjaro regions. For instance, in November 2016, anthrax outbreaks were reported in Monduli district, Arusha region in Northern Tanzania in which 131 carcasses of wild animals were disposed of and 39 carcasses of domestic animals were reported to be consumed [18]. In the Serengeti ecosystem of Northern Tanzania, serological reactions have been reported in herbivorous species often hunted for bushmeat that comes from wildlife which is smuggled in for human consumption [26]. Spillover infections in wildlife can sustain the disease and become a source of spill-back infection to humans and livestock [27].

Therefore, recurrent outbreaks of anthrax in Northern Tanzania are probably due to the extensive interactions of human, livestock and wildlife in the interface areas. Sporadic, non-fatal cutaneous anthrax lesions are common in individuals who handle infected meat or come in direct contact with infected animal materials [18]. Although it is well known that cutaneous anthrax is caused by skin contact with contaminated surfaces [28], during these outbreaks it was not clear which surfaces were the most important vehicle for transmitting *B. anthracis* to humans in specific geographical and cultural settings.

Other studies have reported that there is limited knowledge on the community's awareness of the role contributed by the interaction of animals and humans in the transmission of zoonotic diseases [29,30]. Our retrospective study of health facilities and animal diagnostic centres from 2006 to 2016 revealed a list of hotspot districts for anthrax outbreaks in Northern Tanzania, and that most reported human cases pertained to cutaneous anthrax infection [16]. Moreover, the Arusha region had a reported incidence of 7.9 human anthrax cases per 100 000 population followed by the Kilimanjaro region with 6.6 per 100 000 population [16].

During anthrax outbreaks, the multisectoral teams comprising experts from the ministries responsible for human, livestock and wildlife health were dispatched to the affected regions. In these affected areas, a team collaborated with the regional and district's multisectoral teams to contain the outbreaks by mounting preventive and control measures including intensified surveillance, community awareness, improved diagnostic capacity and livestock vaccination against anthrax in the affected areas [31]. In Tanzania, the coordination of response to disease outbreaks is under the One Health coordination desk within the Prime Minister's Office [32,33].

The current study was conducted to identify demographic and behavioural factors associated with cutaneous human anthrax outbreaks in the anthrax hotspot areas of Northern Tanzania. The study was conducted to better understand the causal relations and improve on potential intervention strategies in the region.

## 2. Material and methods

### 2.1. Study area

**Figure 1.**
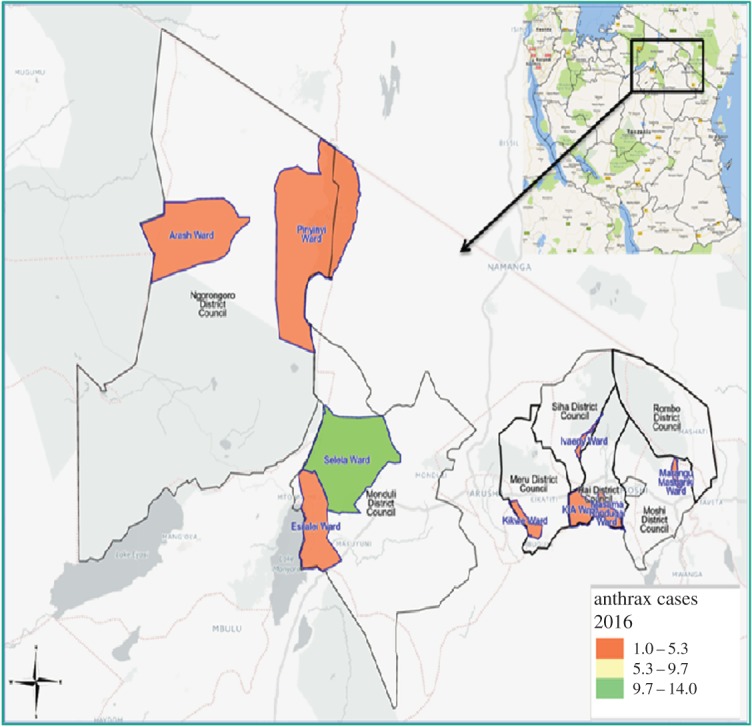
Spatial distribution of anthrax cases in the affected wards from hotspot districts, Northern Tanzania 2016.

The areas for this study were the hotspot districts for anthrax in the Arusha and Kilimanjaro regions of Northern Tanzania. The eligible districts for the Arusha region were Ngorongoro, Monduli and Meru, while in the Kilimanjaro region the study was conducted in Siha, Hai, Rombo and Moshi rural districts. The health facilities involved in each district included Wasso DDH, Endulen Mission Hospital, Pinyinyi, Piyaya, Arash and Magaiduru Dispensaries in Ngorongoro District. Selela, Oltukai, Mto wa Mbu, Mungere, Mbaash and Mswakini Dispensaries were the studied health facilities in Monduli District, while Majengo Dispensary was in Arumeru District. Other health facilities in the study were Hai District Hospital, Sanya Station, KIA and Mtakuja Dispensaries in Hai District; Himo, Rauya RC Dispensaries in Moshi rural district, while Kibong'oto Hospital and Manyata Dispensary were in Siha District. Lastly, Huruma DDH, Nanjara and Karume HC were included in Rombo District. Figure 1 shows the wards where the health facilities and villages involved in the study are located. All the studied districts have a majority of residents practising both subsistence farming and animal husbandry. The study districts are also in the interface areas surrounded by different wildlife conservation areas in the northern circuit of Tanzania. There are soft/porous borders between wildlife conservation areas and human settlements, due to an increased interaction between wildlife and livestock during grazing and at water points [34] in Northern Tanzania. Humans are also posing a risk of zoonotic disease transmission through farming intensification in close proximity to conservation areas, leading to clearance of bushes (change of landscape) and hence destruction of the wildlife ecosystem, causing an increased rate of contact between disease pathogens and humans, livestock and wildlife [35,36]. The data collection for this study was done from 6 October to 5 December 2016.

### 2.2. Study design and sample size

This study was of a non-matched case–control design, with cases being retrieved from the local health facilities. For each case, a control was selected from a nearby randomly selected household within the same locality as the eligible case.

The minimum sample size was calculated using the Epitools AusVet sample size calculator (http://epitools.ausvet.com.au/content.php?page=SampleSize) with the assumption that the frequency of exposure in controls was 20%, and the odds ratio was to be detected at 3.0, with 80% power and a 95% confidence interval. With these assumptions, the minimum sample size of 61 cases and 61 controls was calculated. A total of 59 cases and 59 corresponding controls were subsequently recruited.

### 2.3. Inclusion and exclusion criteria

A case was defined as any person residing in the selected hotspot districts of Northern Tanzania who had ever developed skin lesions by itching of the affected area followed by papular lesions and thereafter a vesicular stage over 2–6 days, eventually developing into depressed black eschar sometimes accompanied by mild or severe oedema [1]. A case was eligible for inclusion in the study if records were found in the medical register at a randomly selected health facility in the hotspot districts of the Arusha and Kilimanjaro regions during the preceding two weeks. The patient should have met the case definition for cutaneous anthrax (as defined above) and his/her name found registered in medical records and had resided in the hotspot districts of the Arusha and Kilimanjaro regions for not less than six months before the time of recruitment. A control was defined as any person who resided in a neighbourhood with an eligible case and had not contracted cutaneous anthrax during the preceding six months. This study excluded anthrax suspected cases with a history of coming from other places apart from the Arusha and Kilimanjaro regions in a period of one week before the onset of signs and symptoms of anthrax. Children under 18 years old were included in the study, but their parents/guardians were interviewed as a proxy on their behalf.

### 2.4. Data collection

A semi-structured questionnaire was developed in English to be administered to both the cases and the controls. The questionnaire included questions related to potential biological exposure to *B. anthracis* as well as information about demographic factors such as age, sex, occupation, ethnic group, level of education, district/place of residence, and potential risks linked to travelling outside the village in the last two weeks before onset of the disease. The questionnaires were pretested, and necessary changes were made based on the identified ambiguities. The questionnaire was subsequently translated into Kiswahili, the national language spoken by almost every resident.

Before visiting the eligible households, a brief interview was conducted with the ward and village executive officials. Locally available public health officers, livestock extension officers and natural resources officers were also interviewed to document their views on the occurrences of the human and animal anthrax cases in their areas within a period of one month before the time of data collection.

In each household of an eligible human anthrax case, interviews were conducted using the questionnaire and in the event of an underage case (less than 18 years), the proxy (parent or guardians) was interviewed in the same household. After the case interview, the questionnaire was administered to the head of households near the cases, which served as the control.

### 2.5. Statistical methods and data analysis

The data obtained were entered into a Microsoft Excel^®^ spreadsheet by allowing comparison for duplicate data entry errors, and data cleaning was done to ensure the quality of the information entered in the dataset. The cleaned dataset was then transferred into STATA (Stata15/SE for Windows, StataCorp, College Station, TX, USA) for statistical analysis.

Essential demographic, biological and other characteristics were described for cases and controls. The relationship between anthrax transmission and potential risk factors or other covariates was initially assessed using univariable logistic regression. As many cases were younger compared to the controls, the analysis was split into four age quantiles. Further recoding of all exposure factors as dichotomous (yes/no) variables was done. Candidate variables with *p* < 0.25 from the initial logistic models were subsequently assessed for collinearity in a cross-tabulation using a Goodman and Kruskal's gamma test. For highly correlated variables, only one of them was selected for further analyses. Variables were identified as confounders and included in the final model if including or excluding the variable altered the effect estimate for another variable by more than 10%.

The first statistical model was developed using a multivariable logistic regression, with a backward elimination strategy among candidate variables. The models were built based upon the Wald test and the likelihood test (*p* < 0.05). We finally used a Hosmer–Lemeshow test for the goodness of fit and the area under the curve of the receiver operating characteristics to assess the reliability of the final constructed model.

As many exposure variables were correlated, we were faced with the difficulty in establishing a realistic and stable statistical model. Considering the questionnaire used in this study, we noted that there were groups within our investigated population. Those groups were characterized by different patterns of behaviour, caused by disparate preferences, which could lead to anthrax infection. Still, we could not identify any specific variable which describes such behavioural dichotomy. Hence, we adjusted our statistical analyses using a latent class analysis (LCA) method. All variables linked to the expected exposures to anthrax were used to construct two latent classes using the generalized structural equation modelling (*gsem*) command in STATA*.* The binomial family and the logit link function defined the variables. The study subjects were classified with a probability of belonging to an *Exposed* class and the rest as *Not Exposed* by using a posterior probability of greater than 0.5 as a cut-off/threshold between the two classes. Based on a directed acyclical graph (DAG) model drawn in the DAGitty software [37], the final statistical model was established using a structural equation model (SEM). The SEM was also built on the *gsem* platform with a logit link function between the anthrax cases and the *Exposed* class*.* Initially, the primary model was built using the graphical interface in the *sembuilder*, before modifying the model in the *gsem* command syntax. Demographic factors such as age, sex, occupation and education as well as the history of travel were used as predictors for *Exposed* and were not linked directly to anthrax cases. As there was a strong age bias in the dataset due to the high number of young cases, separate SEM models for the first age quantile (less than 20 years) and older study subjects were established.

## 3. Results

### 3.1. Respondents’ characteristics

**Figure 2.**
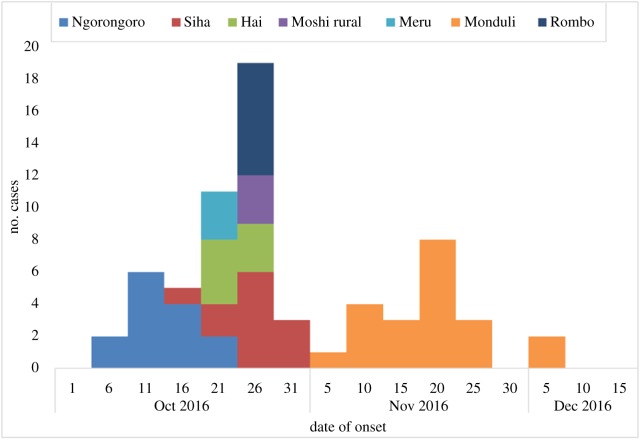
The cumulative epidemic curve for identified anthrax cases in the hotspot districts of Northern Tanzania in the period of October–December 2016.

**Figure 3.**
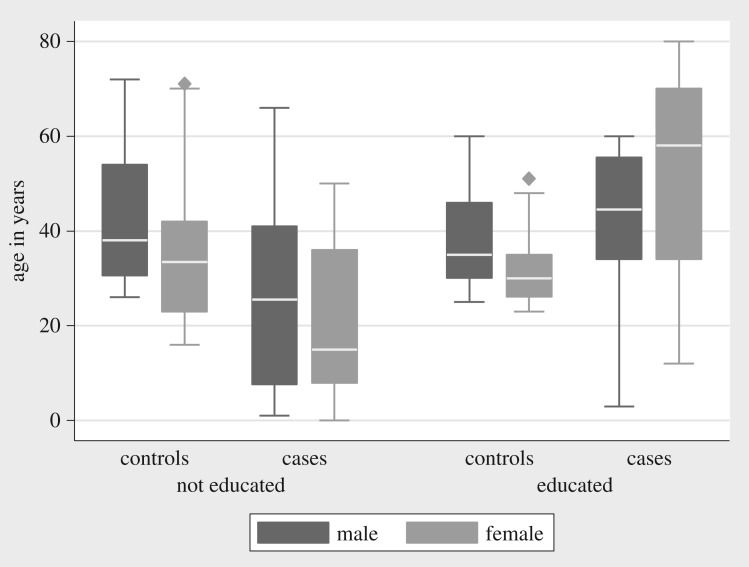
Demographic characteristics of study subjects in the hotspot districts of Northern Tanzania, 2016.

Cases were recruited from Hai (*n* = 6, 10.2%), Meru (*n* = 3, 5.1%), Monduli (*n* = 20, 33.9%) Moshi DC (*n* = 3, 5.1%), Ngorongoro (*n* = 12, 20.3%), Rombo (*n* = 7, 11.9%) and Siha (*n* = 8, 13.5%). Figure 1 illustrates the relative density of cases recorded in each of the wards from the hotspot districts. The timeline for the cases recorded in the different districts is found in figure 2. Table 1 gives the main categories of the demographic and biological variables recorded. Among the study participants, there were more male (*n* = 70, 59.3%) than female participants (*n* = 48, 40.7%). The age range of participants was 1–80 years with a median age of 32 years. Figure 3 shows the distribution of age across cases and education groups. A total of 83 (70.3%) of the study subjects had no formal education. During analysis, it was realized that younger cases (1–20 years) were more recruited, with 26 (44.1%) of the 59 cases, while only four controls (6.8%) were from this group.

**Table 1.** Univariable logistic regression analysis of demographic, biological and other risk factors associated with anthrax transmission, Northern Tanzania 2016. Results are given as the OR with the corresponding *p*-values.

variable	variable description, *n* (%)	dataset, OR (*p*-value)	age quantiles (years)			
			1–20 OR (*p*-value)	21–30 OR (*p*-value)	31–40 OR (*p*-value)	≥41 OR (*p*-value)
demographic characteristics						
education	some education, 35 (30)	0.4 (0.02)^a^	6 (0.001)^a^	0.1 (0.05)^a^	0.4 (0.32)	2.1 (0.31)
	not educated, 83 (70)					
sex	male, 70 (59)	0.8 (0.70)	3.0 (0.06)^a^	0.5 (0.5)	1.3 (0.70)	0.9 (0.9)
	female, 48 (41)					
occupation	risky, 112 (95)	1.0 (1.00)	omitted	omitted	omitted	omitted
	not risky, 6 (5)					
biological factors						
skinning/burying	yes, 75 (64)	1.6 (0.18)^a^	3.3 (0.27)	0.7 (0.74)	0.6 (0.53)	4.2 (0.09)^a^
	no, 43 (36)					
contact with livestock	yes, 78 (66)	6.1 (0.00)^a^	2.5 (0.47)	3.8 (0.14)^a^	14.6 (0.01)^a^	3.1 (0.18)^a^
	no, 40 (34)					
contact with animal products	yes, 78 (66)	6.1 (0.00)^a^	12.0 (0.04)^a^	4.6 (0.09)^a^	4.4 (0.10)^a^	3.1 (0.18)^a^
	no, 40 (34)					
history of travel	yes, 9 (8)	1.2 (0.72)	omitted	omitted	omitted	0.4 (0.50)
	no, 109 (92)					
sleeping materials	mattress, 64 (54)	2.6 (0.01)^a^	5.5 (0.13)^a^	3.25 (0.15)^a^	1.3 (0.71)	0.5 (0.56)
	animal skins, 54 (46)					
other variables						
source of animal feeds	risky, 82 (69)	0.6 (0.02)^a^	0.3 (0.16)^a^	0.81 (0.66)	0.7 (0.4)	0.9 (0.8)
	not risky, 36 (31)					
knowing animal's vaccine preventable diseases	yes, 30 (25)	0.3 (0.04)^a^	0.7 (0.7)	omitted	0.27 (0.27)	1.3 (0.74)
	no, 88 (75)					
animal died at compound	yes, 65 (55)	13.16 (0.00)^a^	75.0 (0.01)^a^	14.8 (0.01)^a^	28.8 (0.00)^a^	1.5 (0.56)
	no, 53 (45)					
disposal of animal carcasses	consume, 64 (54)	14.37 (0.00)^a^	75.0 (0.01)^a^	14.8 (0.01)^a^	28.8 (0.00)^a^	2.13 (0.33)
	not applicable, 54 (46)					
source of meat	home slaughter, 71 (60)	1.9 (0.09)^a^	2.5 (0.4)	1.8 (0.40)	1.4 (0.6)	0.6 (0.5)
	from the butcher, 47 (40)					
keep animals	yes, 94 (80)	3.87 (0.01)^a^	8.3 (0.16)^a^	1.75 (0.54)	3.6 (0.26)	2.6 (0.31)
	no, 14 (20)					
keep dogs	yes, 73 (62)	3.5 (0.002)^a^	25 (0.02)^a^	3.8 (0.14)^a^	1.6 (0.52)	1.2 (0.8)
	no, 45 (38)					

^a^Candidate variables.

### 3.2. Logistic regression analysis

In the univariable logistic regression analysis, study subjects were initially grouped into four age quantiles and subjected to cross-tabulations with demographic characteristics (sex, education status and occupation) and history of travel and biological factors (skinning/burying dead animal, contact with animals, contact with animal products and type of sleeping materials). These are the factors which may predispose an individual to anthrax infection; the other variables were source of animal feeds, knowledge on the animal diseases preventable with vaccine, disposal of animal carcasses, death of animals at home and keeping animals/dogs. Potential candidate variables (*p* < 0.25) are presented in table 1. The results from the subsequent multivariable logistic model are presented in table 2. These results indicate that having primary school education was protective against getting anthrax infection (OR = 0.02). There was no association between knowledge of disease prevention through vaccination or of anthrax as a disease. Increasing age, 21–30 years (OR = 0.07) and 31–40 years (OR = 0.08) were protective against acquiring anthrax infection compared with the younger group, 1–20 years. A worrying sign was that the biologically relevant variables such as skinning infected animals, touching the infected animals and their products, and sleeping on the infected animal's skin, which were linked to direct exposure to anthrax infection, disappeared from the multivariable model.

**Table 2.** Results from multivariable logistic regression analysis for potential predictors associated with anthrax transmission in Northern Tanzania, 2016. Results are given as the odds ratio (OR) with 95% CI and corresponding *p-*values.

variable description	OR (95% CI)	*p*-value
age 1–20 years	1.00 (–)	—
age 21–30 years	0.07 (0.011–0.47)	0.006
age 31–40 years	0.08 (0.016–0.43)	0.003
age ≥40 years	0.56 (0.13–2.43)	0.445
no formal education	1.00 (–)	—
have formal education	0.02 (0.0024–0.16)	<0.001
does not know animal diseases preventable by vaccines	1.00 (–)	—
know animal diseases preventable by vaccines	0.23 (0.055–1.02)	0.053

### 3.3. Latent class analyses

**Figure 4.**
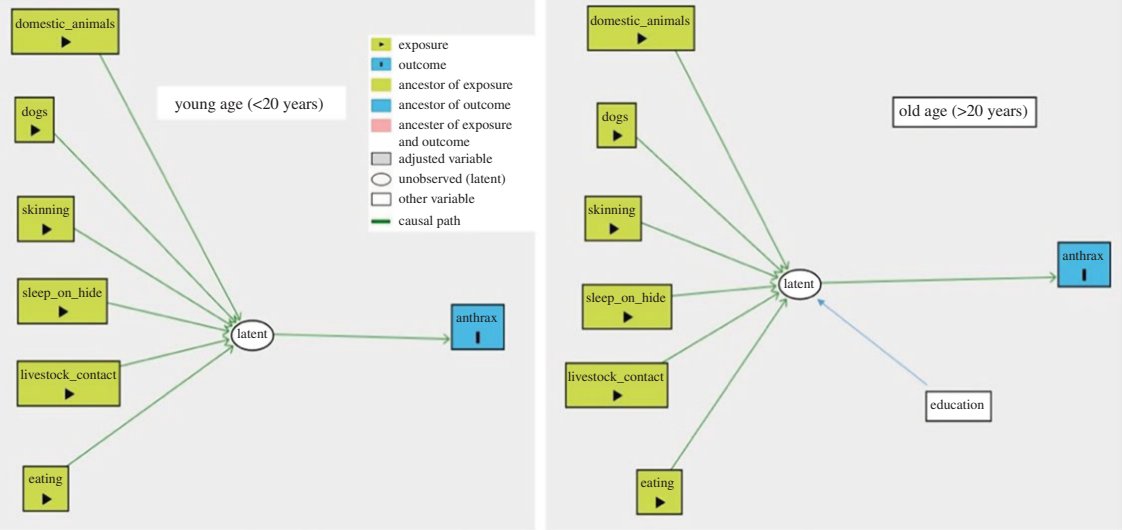
Direct acyclical graph (DAG) for the suggested causal pathway of anthrax transmission in the hotspot districts of Northern Tanzania, 2016.

The LCA model was used to shift the focus from simple associations in the multivariable analysis model to describing a potential causal pathway of the exposures and anthrax infection with age and education being used as primary variables, based upon the assumed causal diagram shown in figure 4. The level of education was not considered for the youngest group (1–20 years), as they are not eligible for enrolment in primary education, which mostly starts at the age of 7 years or/and above. The LCA showed that 73/118 (61.9%) of the study subjects had a high probability of being classified as exposed, while 45/118 (38.14%) of the study subjects were in the unexposed group. As there was a strong age bias among cases, two final SEMs: one with the youngest (1–20 years) group and one for the older group (greater than 20 years) were established. In the youngest group, exposure status was strongly linked to anthrax transmission (OR = 25.0, 95% CI = 1.5–410). In the older group, the link to exposure was smaller but still high (OR = 3.2, 95% CI = 1.28–8.00). The most distinct difference between the SEM model and the ordinary logistic model was that we were able to identify that education was linked to the model as a predictor of exposure, but not directly to anthrax infection (table 3).

**Table 3.** SEM for predictor variables for anthrax transmission in the hotspot districts of Northern Tanzania, 2016. Results are given as the OR with 95% CI and the corresponding *p*-values.

variable description	age 1–20 years, OR (95% CI); *p*-value	age greater than 20 years, OR (95% CI); *p*-value
not exposed (LCA class)	OR = 1.00 (–)	1.00 (–)
exposed (LCA class)	25.0 (1.5–410); *p* = 0.024	3.2 (1.28–8.0); *p* = 0.013
no formal education	—	1.00 (–)
have formal education	—	0.23 (0.09–0.58); *p* = 0.002^a^

^a^Having formal education was a predictor for being exposed, not directly linked to skin anthrax.

## 4. Discussion

The occurrence of anthrax outbreaks in a particular location mostly depends on the existence of interacting factors, which include unique characteristics of the bacterium, environmentally related features, animal densities and human activities [9,38].

In this study, we found that some human activities predisposed people to risk factors for cutaneous anthrax infection. For the younger group (less than 20 years), we only found a strong relationship (OR = 25) between the set of exposures measured as a latent class representing many exposures and cutaneous anthrax. In the older group (age greater than 20 years), we still found a considerable risk as measured by an OR of 3.1 among the exposed. However, using the SEM, we could explain the probability of belonging to the *exposed* class by the education level. Thus, factors such as knowledge of animal diseases preventable by vaccines found in the ordinary multivariable model were found to be proxy variables better represented by the level of education. We applied a strict causal understanding in establishing the SEM, especially benefitting from using the latent class to represent a series of correlated exposure variables. The exposure variables discussed below all disappeared as risk factors using the standard multivariable approach. The neglect of biological plausibility in statistical analyses, during epidemiological studies such as this, is an inherent issue [39], which we sought to address in this study. Activities such as keeping livestock, skinning dead animals, coming into contact with animals or their products, touching carcasses and burying carcasses were in our analyses set as exposure variables for disease occurrence. The nature of livestock rearing within these households brings about a high chance of direct/indirect contact with contaminated animals or animal by-products, which makes these factors pivotal to a high predisposition of humans to acquiring cutaneous anthrax [17].

In the pastoral Maasai community, animal skins are mostly used as bedding materials. The use of skin from infected animals would facilitate direct contact with *B. anthracis* spores if the animals had died of anthrax. Other studies have also reported that processing skin and hides for making sleeping materials facilitated anthrax transmission in a susceptible population [40].

This study also found that dressing of dead livestock and using them as meat for human consumption was a risk factor for disease transmission. On some occasions, dead and decomposed animals were found lying on the grounds and being eaten by scavengers. This may be another pathway that may have contributed to an increased disease transmission to the unaffected animals and humans in the hotspot areas. Other studies report that burning, or burying followed by disinfection of the burial site, is an appropriate disposal mechanism for anthrax carcasses and limits further spread of the disease in the affected areas and beyond [18].

In a study done in Zambia, people who participated in skinning infected carcasses and processing meat and hides as well as skins for making sleeping materials were highly exposed to anthrax infection [41]. Another study from Lake Rukwa valley in southwest Tanzania reported that touching infected carcasses and animal products was a potential risk for anthrax transmission in the community [20].

Age and gender distribution can also facilitate disease transmission in a society due to the allocation of duties among the members of the Maasai community. In our study, we found that men of older age were more often affected by cutaneous anthrax, perhaps because they are the ones responsible for taking care of the animals while grazing. In addition, they are also dealing with milking, slaughtering and skinning the carcasses.

Illiteracy was at high levels among the study subjects, and in anthrax cases in particular, and education level was found to be a critical factor in explaining the set of exposure variables. Illiteracy is linked to poverty, and poor people opt to dress a carcass and use it as meat, which exposes them to *B. anthracis* infection by contact in cases of infected animal products [29]. The uneducated part of the community also has trouble following or understanding critical messages through written materials (leaflets, billboards and magazines), which are provided during health education campaigns. Another study reported that poverty is centred in sub-Saharan Africa where most people are illiterate, and the community is predisposed to many infectious zoonotic diseases due to their increased contacts with animals, yet with limited access to good health services for humans and animals [5].

Keeping livestock for the livelihood of pastoral and agro-pastoral Maasai communities is a common practice in Northern Tanzania. However, the animal husbandry systems do not take into account the prevention of animal diseases like anthrax. Because of extensive grazing of livestock, they frequently come into contact with carcasses of wild animals, which may have died from anthrax [42]; this increases the risk of human–pathogen interaction [40]. This is because the pastoral and agro-pastoral communities have intimate contact with livestock in their daily cultural practices as part of a unified social and ecological context [43]. Therefore, social factors and cultural practice dimensions influence the human and animal interactions that propagate the transmission of anthrax in this community [44]. Similarly, researchers have to consider immutable beliefs and cultural practices, which exacerbate risks for transmission of zoonotic diseases, in order to advocate for adequate individual behaviour change [44,45]

In the pastoral and agro-pastoral communities, it is a common practice to keep dogs for security purposes against wild animals and to assist in the herding of livestock in grazing areas. Previous reports found a high seroprevalence of anthrax in dogs owned by pastoralists during a large anthrax outbreak in livestock in the Ngorongoro Conservation area, Northern Tanzania [26].

## 5. Conclusion

Sleeping on animals' skins and contact with infected carcasses through skinning or butchering were linked to the *exposed* latent class, which proved a robust predictor of anthrax infection. For older participants (greater than 20 years), being exposed to the pathogen could be explained by the level of education; where a lack of a formal education was linked to higher risks of anthrax infection. Prevention and control strategies of anthrax in pastoral and agro-pastoral Maasai communities need a well-framed approach with a clear understanding of social mechanisms. Educational materials will need to breach the high levels of illiteracy and provide a socially relevant context.

Therefore, preventive and control measures of anthrax outbreaks such as livestock vaccination, safe carcass disposal (preferably incineration) of dead animals from anthrax, public awareness campaigns, stockpiling of antibiotics, identification of competent laboratories for human and animal anthrax diagnosis, and an intensified surveillance system for human and animal sectors should be implemented using the One Health approach in the hotspot districts of Northern Tanzania. The collaboration of anthropologists and veterinary, medical and public health professionals could bridge the education gap in this unique African environment.

## Supplementary Material

Transmission routes for human cutaneous anthrax outbreaks in the hotspot areas of northern Tanzania. The cutaneous anthrax outbreaks are exacerbated by the increased contact with the infected animals and animal by-products, poor farming practices and mismanaged movement of animals in the disease hotspot areas of northern Tanzania. Moreover, the human behavior of consuming raw or undercooked carcasses from sick or dead animals, poverty and lack of a formal education especially in the Maasai community of Tanzania are the main contributing factors for frequent outbreaks in these areas. Therefore, strategies for prevention and control of this should involve human, livestock, and wildlife sectors in a One Health approach

## Supplementary Material

Interactions between livestock-wildlife and humans as a facilitating factor for continued transmission of B. anthracis in the hotspot areas of northern Tanzania. In our study, we have demonstrated that there was a close temporal relationship between the occurrence of anthrax outbreaks in animals (livestock and wildlife) and in humans. This might have been attributed by the existing interaction between the contaminated environment, livestock, wildlife, and humans. The role of scavengers like stray dogs consuming on the infected carcasses can be some of the facilitating factors. For transmission of anthrax in the places where anthrax outbreaks have occurred are being facilitated by sharing of water collected points between humans and animals, an increased interaction of wildlife and livestock during grazing, and poor disposal of anthrax-infected animal carcasses.

## Supplementary Material

Control measures of anthrax outbreaks in the hotspot areas of northern Tanzania. One of the recommended control measures of anthrax outbreak is safe disposal of carcasses. Either burying or burning of carcasses depending on the physical condition (fresh or dry) of the carcass can help to attain this intervention. Burying can only be considered when the carcasses are fresh and intact because B. anthracis spores can remain virulent for many years under favorable soil condition and become source of future outbreaks when exposed to aerobic environment. The burial pit should be 6 feet deep and lime has to be poured on top of the pit and the surrounding environment in order to decontaminate the location. This method is tedious and labor intensive, as it requires manpower to excavate the burial pit and collection of carcasses into the pit. In contrast, incineration of the carcasses (fresh or dry) should be prioritized as one of the best and most effective control measure during anthrax outbreaks in which fire woods and petrol can be required.

## Supplementary Material

Regular health education as a tool for controlling anthrax outbreaks in the hotspot areas of northern Tanzania. Furthermore, health education is paramount for control of disease spread during anthrax outbreaks. Important and targeted messages are aired to the affected communities through public address, leaflets, and use of the most respected local leaders like ‘the Laigwanan’ in the Maasai community. These leaders are more influential to the society so the messages given by them are highly trusted and comprehended by their community members. The key messages provided includes: (i) don't open the carcass of animals suspected to have died from anthrax because by doing so, you expose the spores into the aerobic environment and they can sporulate and cause more outbreaks, (ii) don't eat raw and/or undercooked meat from dead animals, (iii) take your animals for vaccination depending on the schedule given by your local veterinary expert and, (iv) when fall sick go to the neighboring health facility for medical attention.

## Supplementary Material

Distribution of anthrax cases per village in the hotspot districts of northern Tanzania

## Supplementary Material

Elibariki Mwakapeje Figure6 Dataset Case Control study

## Data accessibility

The datasets supporting this article have been uploaded as part of the electronic supplementary material.
