# Supplementary material for: Risk factors for human cutaneous anthrax outbreaks in the hotspot districts of Northern Tanzania: an unmatched case–control study
Source: R Soc Open Sci. 2018 Sep 5;5(9):180479. doi: 10.1098/rsos.180479 (PMC6170534; doi:10.1098/rsos.180479)
Supplement: Transmission routes for human cutaneous anthrax outbreaks in the hotspot areas of northern Tanzania. The cutaneous anthrax outbreaks are exacerbated by the increased contact with the infected animals and animal by-products, poor farming practices and mismanaged movement of animals in the disease hot [file rsos180479supp1.pdf]

Transmission routes for human cutaneous anthrax outbreaks in the hotspot areas of northern Tanzania

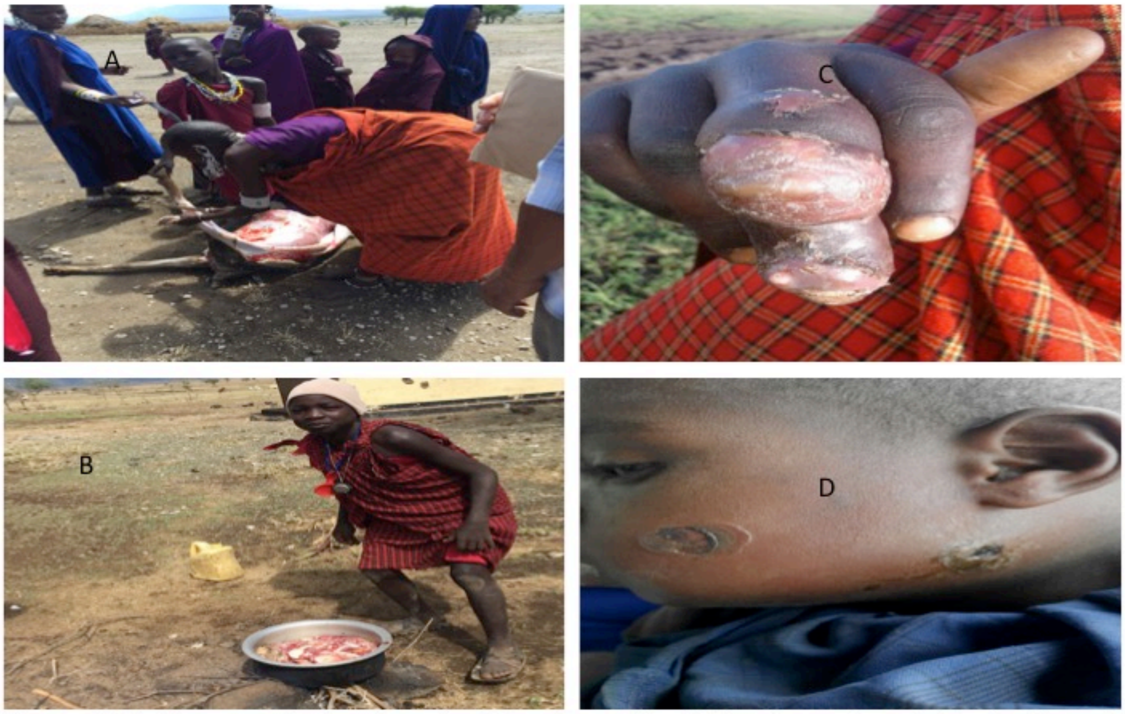

Figure S.1. Photograph showing the Maasai people risking for anthrax infection by – (A) skinning a carcass suspected to die from anthrax, (B) Poor cooking condition of anthrax infected carcass (C) Gangrene of a finger due to anthrax, and (D) Anthrax skin lesions
