# Supplementary material for: Risk factors for human cutaneous anthrax outbreaks in the hotspot districts of Northern Tanzania: an unmatched case–control study
Source: R Soc Open Sci. 2018 Sep 5;5(9):180479. doi: 10.1098/rsos.180479 (PMC6170534; doi:10.1098/rsos.180479)
Supplement: Interactions between livestock-wildlife and humans as a facilitating factor for continued transmission of B. anthracis in the hotspot areas of northern Tanzania. In our study, we have demonstrated that there was a close temporal relationship between the occurrence of anthrax outbreaks in animals (li [file rsos180479supp2.pdf]

Interactions between livestock-wildlife and humans as a facilitating factor for continued transmission of *B. anthracis* in the hotspot areas of northern Tanzania

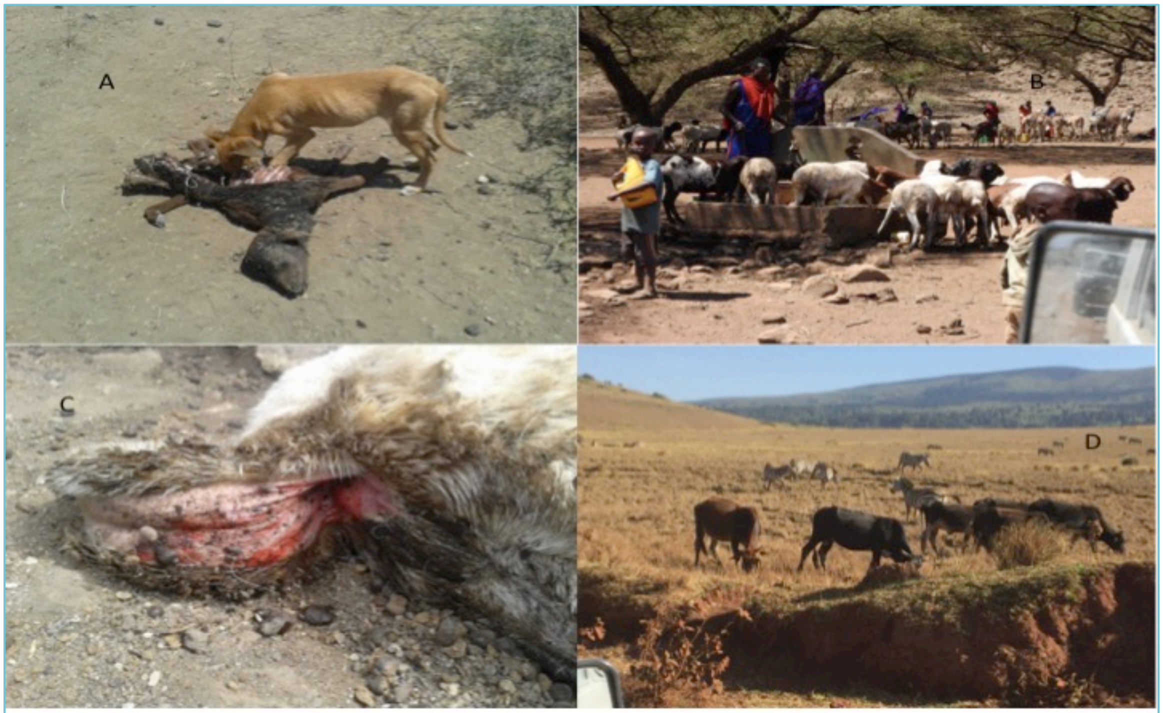

Figure S.2: Photographs showing (A) A dog feeding on abandoned carcass of a sheep (B) animals and humans sharing a water source, (C) Un – attended carcass oozing blood as it was found in one the study sites and (D) livestock and wildlife grazing together in Ngorongoro district, Northern Tanzania
