# Supplementary material for: Risk factors for human cutaneous anthrax outbreaks in the hotspot districts of Northern Tanzania: an unmatched case–control study
Source: R Soc Open Sci. 2018 Sep 5;5(9):180479. doi: 10.1098/rsos.180479 (PMC6170534; doi:10.1098/rsos.180479)
Supplement: Control measures of anthrax outbreaks in the hotspot areas of northern Tanzania. One of the recommended control measures of anthrax outbreak is safe disposal of carcasses. Either burying or burning of carcasses depending on the physical condition (fresh or dry) of the carcass can help to attain this [file rsos180479supp3.pdf]

Control measures of anthrax outbreaks in the hotspot areas of northern Tanzania

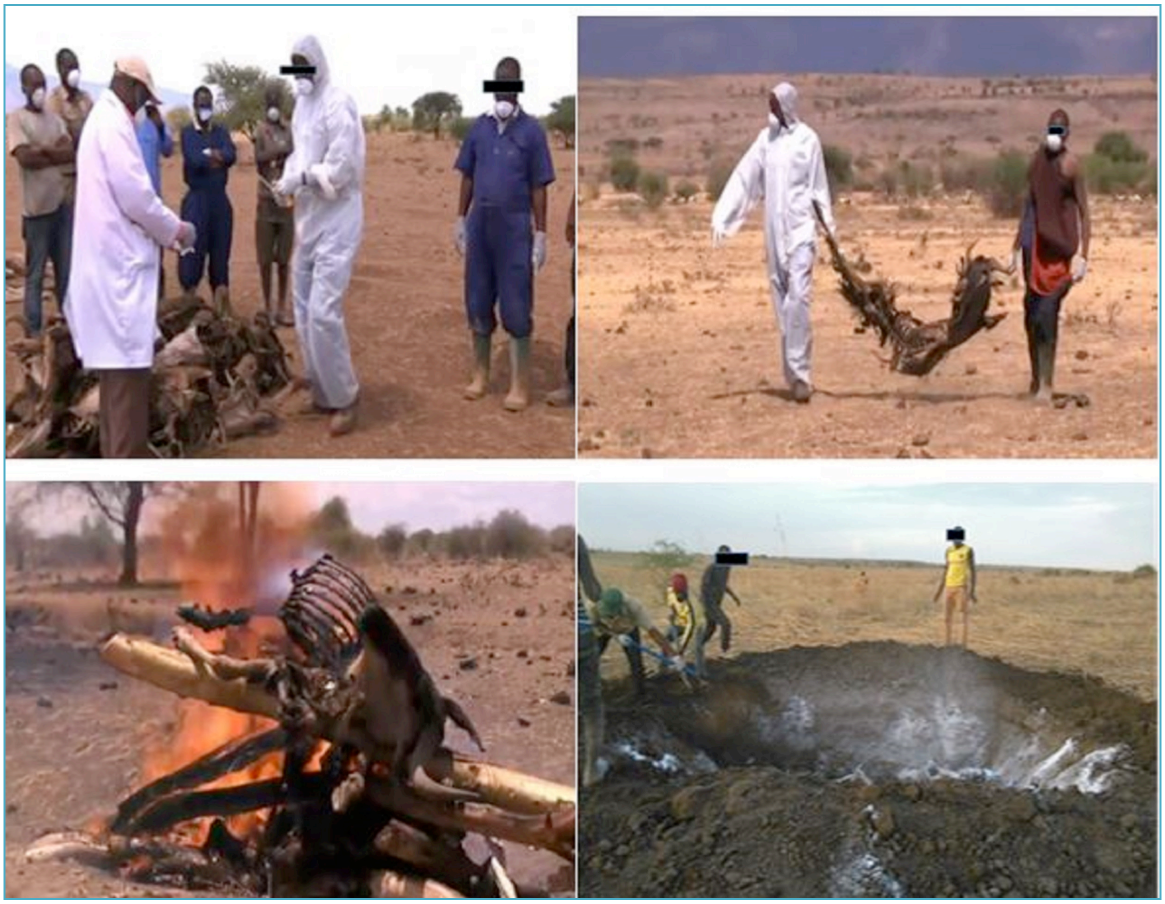

Figure S.3: Options for carcass disposal activities during response to anthrax outbreak in one of the hotspot districts of northern Tanzania.
