# Supplementary material for: Risk factors for human cutaneous anthrax outbreaks in the hotspot districts of Northern Tanzania: an unmatched case–control study
Source: R Soc Open Sci. 2018 Sep 5;5(9):180479. doi: 10.1098/rsos.180479 (PMC6170534; doi:10.1098/rsos.180479)
Supplement: Regular health education as a tool for controlling anthrax outbreaks in the hotspot areas of northern Tanzania. Furthermore, health education is paramount for control of disease spread during anthrax outbreaks. Important and targeted messages are aired to the affected communities through public addr [file rsos180479supp4.pdf]

Regular health education as a tool for controlling anthrax outbreaks in the hotspot areas of northern Tanzania

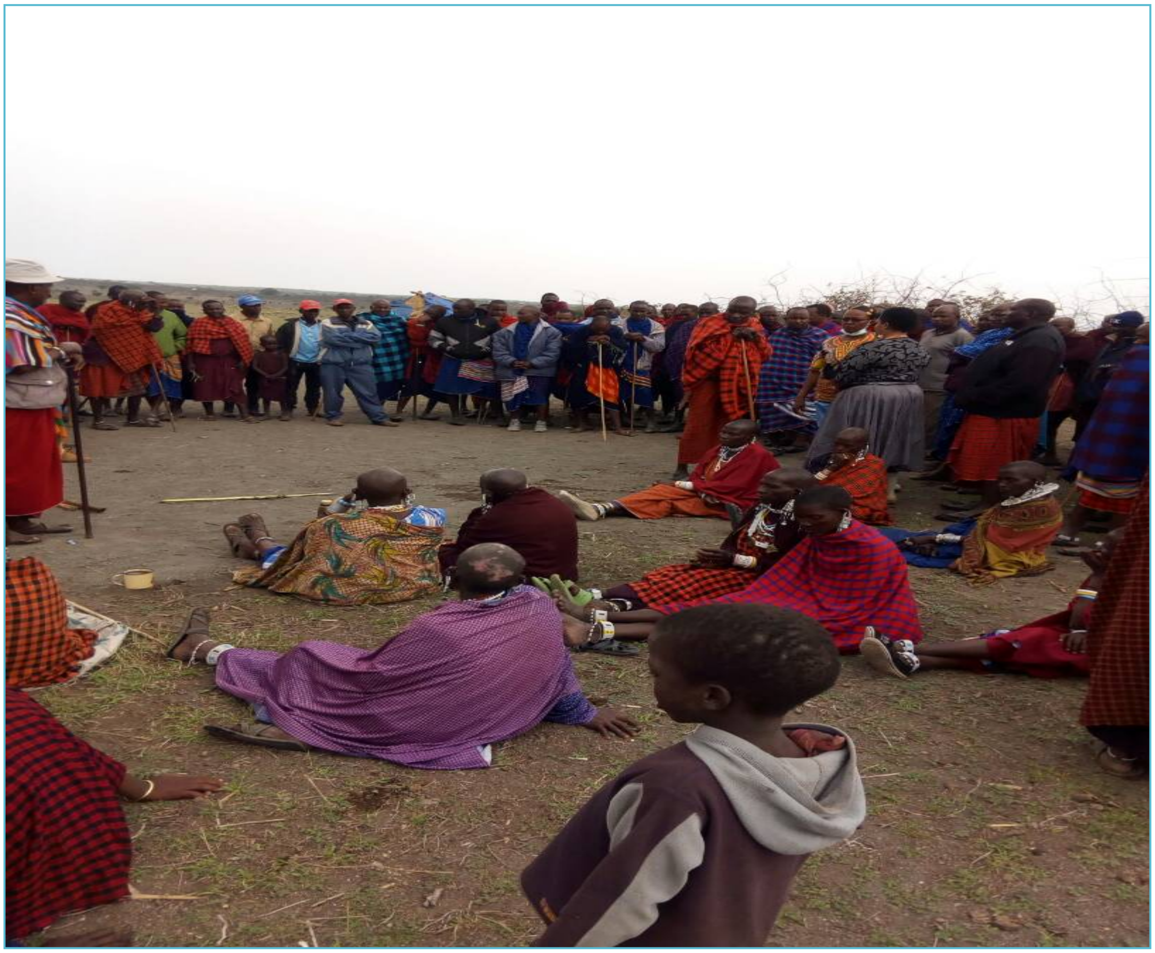

Figure S.4: A well-informed Laigwanan delivering health education to the Maasai community during anthrax outbreak in Monduli district, northern Tanzania
